# Early detection of *Zymoseptoria tritici* infection on wheat leaves using hyperspectral imaging data

**DOI:** 10.1016/j.dib.2025.111404

**Published:** 2025-02-18

**Authors:** Lorraine Latchoumane, Martin Ecarnot, Ryad Bendoula, Jean-Michel Roger, Silvia Mas-Garcia, Heloïse Villesseche, Flora Tavernier, Maxime Ryckewaert, Nathalie Gorretta, Pierre Roumet, Elsa Ballini

**Affiliations:** a AGAP, INRAE, Univ Montpellier, CIRAD, Institut Agro, Montpellier, France; b ITAP, INRAE, Institute Agro, University Montpellier, Montpellier, France; c ChemHouse Research Group, Montpellier, France; d LIRMM, Inria, Univ. Montpellier, CNRS, Montpellier, France; e PHIM, Institut Agro, INRAE, CIRAD, Univ Montpellier, IRD, Montpellier, France

**Keywords:** Plant disease, VNIR, SWIR, Hyperspectral images, Multivariate analysis, Disease monitoring

## Abstract

This article presents a hyperspectral imaging (HSI) database of healthy leaves and leaves infected with *Zymoseptoria tritici* fungal pathogen responsible for leaf blotch (Lb) disease. Leaves of two durum wheat genotypes were studied under controlled conditions to track the evolution of Lb disease and capture significant spectral and spatial differences until the onset of symptoms. Hyperspectral image acquisitions were purchased with two cameras in visible-near infrared (VNIR) and short-wave infrared (SWIR) spectral ranges on eighteen dates between one day before inoculation and twenty days after inoculation. For each wavelength range studied, a total of 1175 images provided information on 3326 leaves measured throughout the experiment. These data are valuable since they can be used as a basis to monitor disease's development over time, to build leaf classification models according to their infection status per genotype per day, to develop prediction models related to symptoms' appearance, or to test imaging and spectral analysis methods.

Specifications Table

**Table:** 

Subject	Analytical Chemistry: Spectroscopy
Specific subject area	Temporal and spectral kinetic of fungal infection of wheat leaves using hyperspectral imaging
Type of data	Raw data: VNIR and SWIR hyperspectral images (.hyspex) and header files (.hdr) Reconstructed images (.jpg) Position of pixels (.csv) Description files (.csv)
Data collection	The experiment was conducted on two genotypes of durum wheat (*Triticum turgidum durum*) cultivated under controlled conditions in a growth chamber. Hyperspectral images were collected *in planta* for twenty-two days to monitor leaf blotch disease caused by the fungal pathogen *Zymoseptoria tritici*. Images were acquired on the first ligulated leaf in a non-destructive way using two devices: HySpex VNIR-1800 and HySpex SWIR-384 (Norsk Elektro Optikk, Norway).
Data source location	Institution: Institut National de Recherche pour l'Agriculture, l'Alimentation et l'Environnement (INRAE) City: Montpellier Country: France
Data accessibility	Repository name: Data INRAE Data identification number: doi:10.57745/WVP0FJ Direct URL to data: https://doi.org/10.57745/WVP0FJ

## 1 Value of the Data

• This dataset depicts the visual appearance and spectral information related to the onset kinetics of Lb disease symptoms on wheat leaves using hyperspectral images acquired post-inoculation.
• The images captured are valuable to monitor the evolution of the Lb disease on wheat leaves through the development of predictive models based on infection status.
• These data can be used to train, test, evaluate, and compare multiple classification models for Lb disease detection at an early stage utilizing the features of hyperspectral images.
• This dataset will improve our insights of the spatial distribution of symptoms associated with Lb disease, enabling researchers to develop phenotyping tools, to optimized farming systems and to guide future experiments.
• Early detection of Lb disease, based on an understanding of host-pathogen interactions, can help reduce losses, prevent the spread of disease and improve overall crop yields.
• The present dataset is of particular interest in various domains such as phenotyping, phytopathology, remote sensing, precision agriculture and chemometrics.

## 2 Background

Wheat (*Triticum turgidum* L.) is the second most widely-cultivated cereal in the world with 803 million tonnes (Mt) produced in 2022 [1]. The European Union is the major producer, with France and Germany the two leader countries growing 34.6 Mt and 22.6 Mt of wheat, respectively [2,3]. The UN-FAO states that to meet global food demand, agricultural production must increase by 50% by 2050. This can be achieved by developing improved cultivars and implementing innovative management practices. To meet future demand, wheat production will need to increase significantly, despite the risks posed by a changing climate to current production rates [4].

Leaf blotch (Lb), previously referred to as Septoria tritici blotch (STB), is the most important and damaging foliar disease on durum wheat (*Triticum turgidum* subsp *durum*) provoked by the pathogenic fungus *Zymoseptoria tritici* (also identified as Mycosphaerella graminicola for sexual stage) [5]. The prevalence of fungal diseases affecting wheat has resulted in a wheat fungicide market in Europe of over 1.3 billion euros, 70% of which is devoted to Lb management [6]. Early detection and protection against diseases are crucial for disease control, crop yield improvement, cost reduction and increased agricultural output. However, disease diagnosis by traditional methods is frequently subjective, laborious and time-consuming. In order to prevent rapid extension of diseases, and thus limit economical losses while reducing chemical fungicide supplies, innovative techniques are developed [7]. Among them, hyperspectral imaging (HSI) offers rapid, cost-efficient and non-destructive analysis capable to detect plant disease at an early state [8,9]. Automatic, more accurate and non-invasive detection will also facilitate and accelerate the large-scale evaluation of new resistant varieties or alternative products to fungicides [10]. HSI has already been applied in laboratory or under in-fied conditions to assess plant spectral and spatial changes in plant when infected [9], including wheat. Indeed, several research studies have been conducted on wheat using HSI for the detection of foliar disease caused by fungal pathogens, such as powdery mildew [11], yellow rust [12] or *Fusarium* head blight [13]. Wheat infection induced by *Z. tritici* was also studied using HSI [[14], [15], [16]]. However, these studies focus mainly on vegetation indices and supervised methods, such as partial least squares discriminant analysis (PLS-DA), support vector machine (SVM) and random forest (RF), among others. Moreover, in our study, measurements have been done on 18 different dates, giving a temporal sampling of the evolution of infected or non-infected leaves using HSI. In the present article, HSI acquisitions were performed using visible-near infrared (VNIR) and short-wave infrared (SWIR) spectral ranges on both healthy and manually infected wheat leaves. This approach has led to original spatio-temporal measurements, combining SWIR and VNIR with imaging for early detection of *Z. tritici* on wheat leaves. These data thus provide relevant information for the study of spectra at the pixel level for supervised and unsupervised models associated with Lb disease on durum wheat leaves.

## 3 Data Description

**Fig. 1:**
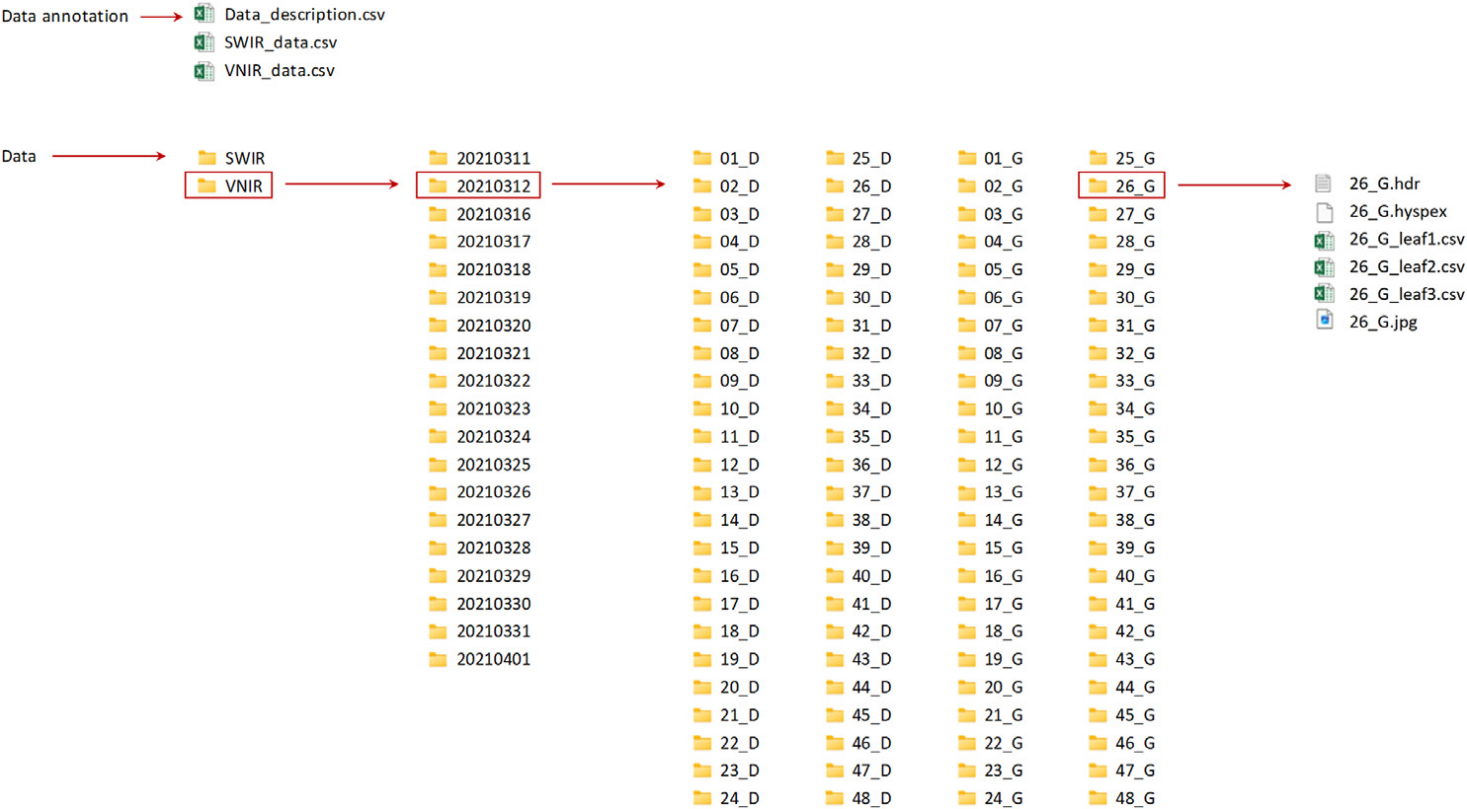
Tree structure of dataset.

The present dataset contains “Data annotation” and “Data” folders. The directory organization structure is shown in Fig. 1. The folder “Data annotation” gathers a description file (.csv) of the whole dataset and two SWIR and VNIR table files (.csv) providing information about the samples and the number of leaves collected per images each day. The folder “Data” contains VNIR and SWIR hyperspectral images in separate “VNIR” and “SWIR” folders. Each of these directories includes 18 compressed folders (.zip) corresponding to the dates on which the images were acquired such as ‘YYYYMMDD’. In these folders, multiple sub-folders are gathered and differentiated according to the label of the samples ‘NUM_SIDE’, with ‘NUM’ corresponding to the number of the plant pot (from 01 to 48), and ‘SIDE’ the measured side of the pot (D for the right side and G for the left side). Images are located in their corresponding sub-folders and stored in ENVI format. Two file types are distinguished for each labelled images: the raw image data (.hyspex) and the metadata file (.hdr) which provides details about the acquisition. The pixels coordinates of each leaf per image are summarized in separated table files (.csv), with the first column indicating the vertical coordinate of the pixel, while the second indicates the horizontal coordinate of the pixel. Finally, an image (.jpg) reconstructed from the hyperspectral image is also contained in related sub-folders.

## 4 Experimental Design, Materials and Methods

### 4.1 Hyperspectral image acquisition

**Fig. 2:**
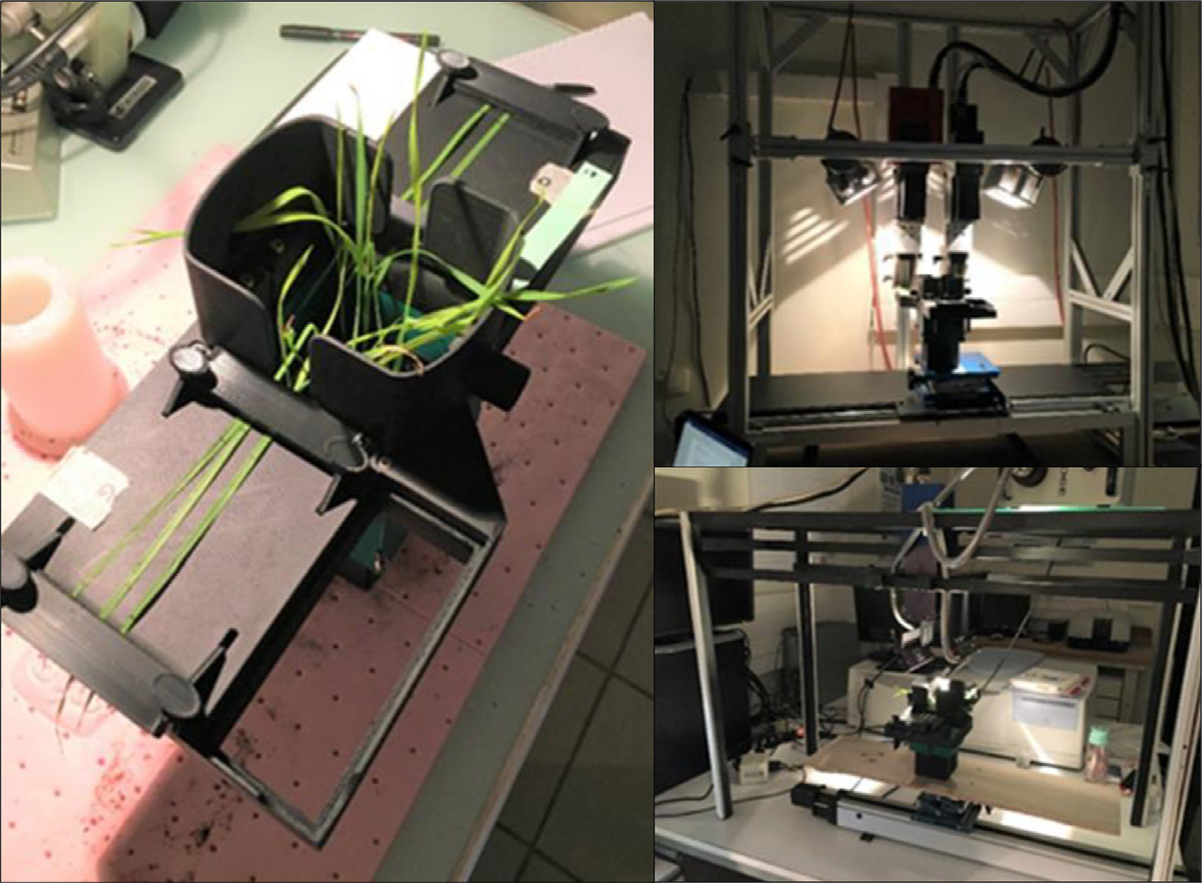
Experimental set-up for HSI acquisitions, showing the leaf arrangement per pot (left), the VNIR camera (top right) and the SWIR camera (bottom right).

Images were acquired on 18 dates using two hyperspectral cameras in pushbroom mode (Fig. 2) to provide images of two-dimensional spatial variables (pixels) and one-dimensional spectral variables (wavelengths). VNIR acquisitions were conducted using the HySpex VNIR-1800 (Norsk Elektro Optikk, Norway), providing 216 wavelength spectra covering a spectral range from 411 nm to 993 nm and a resolution of 1370×1024 pixels, except for the last date which had a resolution of 2740×2048 pixels. SWIR acquisitions were conducted using the HySpex SWIR-384 (Norsk Elektro Optikk, Norway), providing 256 wavelength spectra covering a spectral range from 964 nm to 2494 nm and a resolution of 420×320 pixels. The camera-to-leaf acquisition distance was set at 21.5 cm, with a lens focal distance of 30 cm. Pixel dimensions were 93.4 µm for the x-axis and 87.6 µm for the y-axis for the VNIR camera, while they were 217 µm for the SWIR camera for both x- and y-axis.

Samples were placed on a black support material next to a certified reflectance standard reference (SRS99, Spectralon®) used to normalize spectra and avoid non-linearities in all instrumentation components. For each image, the intensity I(λ) of the reflected light and the intensity I_0_(λ) of the light reflected by the standard reference were measured. The signal without light was represented by the dark current I_d_(λ). For every sample, a reflectance image R(λ) was subsequently computed. We individually adjusted each pixel's reflectance per line as following:

$$R\left(\lambda \right)=\frac{I\left(\lambda \right)-\;I_{d}\left(\lambda \right)\;}{I_{0}\left(\lambda \right)-\;I_{d}\left(\lambda \right)}$$

**Fig. 3:**
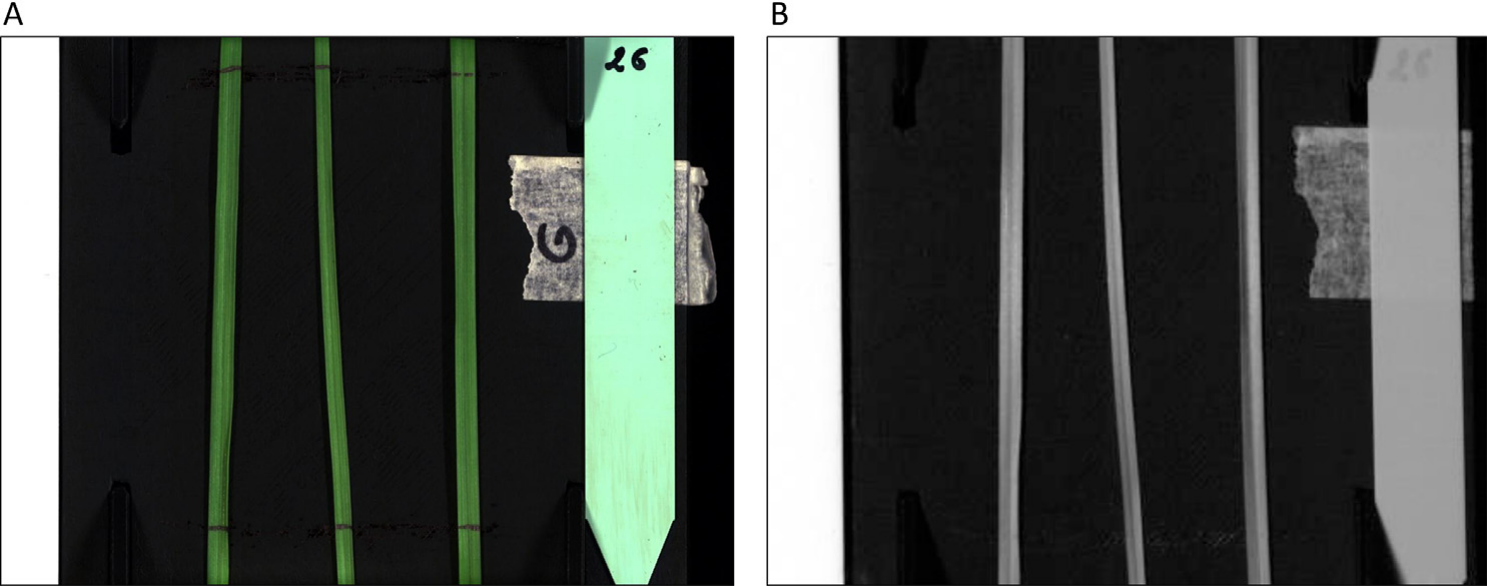
Visualization images from VNIR (A) and SWIR (B) hyperspectral images. A: RGB image was reconstructed using wavelengths 446 nm, 535 nm and 627 nm of VNIR image. B: grey level image was reconstructed using wavelength 994 nm of SWIR image.

Hyperspectral images (.hyspex) directly contain the corrected intensity of the dark current. To enable visualization of the VNIR hyperspectral image, an RGB image was constructed using wavelengths 446 nm (B), 535 nm (G) and 627 nm (R). For SWIR, the monochromatic image at 994 nm was used (Fig. 3).

### 4.2 Biological materials and experimental design

Hyperspectral images were captured on two susceptible durum wheat genotypes originating from the David et al. [17]. Evolutionnary Pre-breeding pOpulation (EPO), *i.e.* EPO_67 and EPO_68. For each genotype, 192 seeds were selected by weighing to ensure that each population was homogenous. The seeds were then sown in 10×10×10 cm pots in Neuhaus S potting soil with pozzolan (5L/70L) supplemented with Flocoat retardant fertiliser (70L/90g of soil). Six plants per pot were arranged in groups of three on opposite right and left sides of the pot. A total of 48 pots were made available: 24 for genotype EPO_067 and 24 for genotype EPO_068. All plants were cultivated in 2021 under controlled conditions in a growth chamber at 23°C with a photoperiod corresponding to 12 hours of light per day [18].

**Fig. 4:**
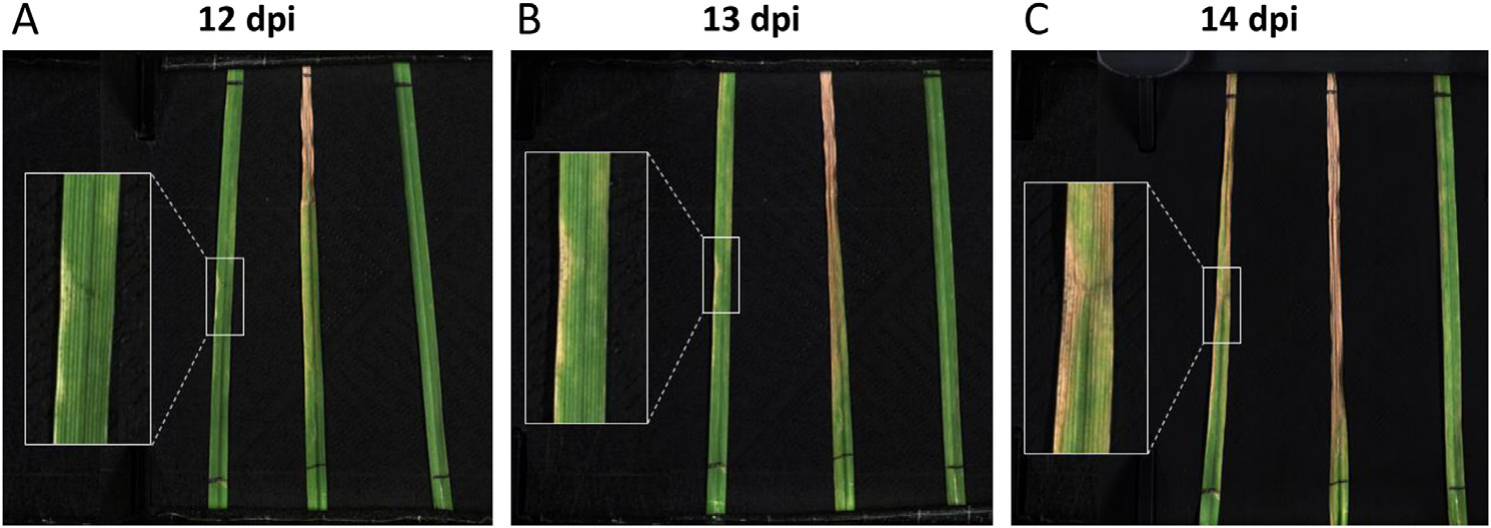
VNIR hyperspectral images (label 45_G) illustrating Leaf blotch disease symptoms of wheat leaves infected with *Zymoseptoria tritici* at 12 dpi (A), 13 dpi (B) and 14 dpi (C). Necrosis and pycnidia (black dots) caused by the disease are highlighted in the white inset.

Wheat leaves infection were performed using the strain P1a of *Z. tritici* responsible for Lb disease [18]. For each genotype, wheat plants of 12 pots, identified as “Infected”, were inoculated with P1a dissolved in distilled water/Tween 20 mixture at a concentration of 1.10^6^ spores/mL, while wheat plants of the other 12 pots, identified as “Non-infected”, were inoculated with distilled water/Tween 20 mixture only. The latter represents the non-infected control, compared to the infected leaves. Inoculation was carried out 14 days after sowing on 8 cm of the adaxial side of the first ligulated leaf using a brush. Inoculation area was delimited using marker. After inoculation, transparent bags were placed on the plants for three days to keep them in high humidity conditions to promote Lb infection. The disease was monitored for up to 20 days post-inoculation (dpi) and symptoms of infection were analyzed by visual inspection throughout the experiment. Symptoms of Lb disease were assessed by the progressive formation of necrosis starting from 7 dpi and the emergence of pycnidia (asexual fruiting bodies) in the final stages of infection (Fig. 4). The expertise of a plant pathologist confirmed the presence of Lb disease at the end of the experiment. In addition, some leaves were sampled on 5 dates (4 dpi, 7 dpi, 10 dpi, 14 dpi and 17 dpi) for biomolecular analyses to ensure that symptoms observed on infected wheat leaves were indeed caused by the inoculated pathogen strain.

**Fig. 5:**
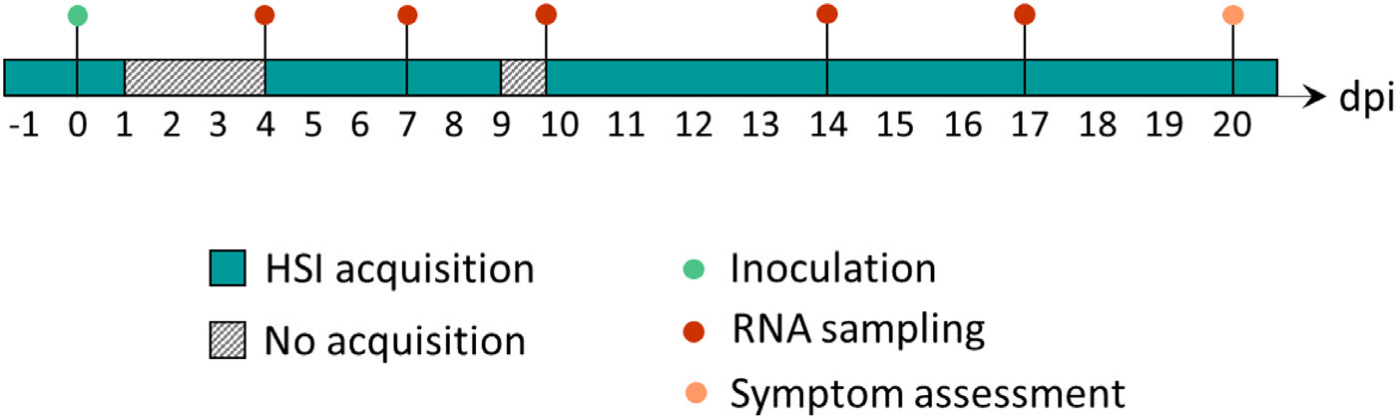
Experimental timetable.

When possible, hyperspectral images were acquired under controlled laboratory conditions on each ligulated leaf of the three individual plants for both sides of the pot. HSI was purchased on 18 dates, starting one day before inoculation and ending 20 dpi (Fig. 5). No HSI acquisition was carried out at 1 dpi, 2 dpi and 3 dpi due to bagging, and at 9 dpi due to technical problems. As previously mentioned, leaf sampling was conducted through the experiment, lowering the number of samples acquired per day as the experiment progressed. In addition, the natural process of senescence, mechanical injuries and/or technical errors contributed to the decrease in sample numbers since they were removed from the dataset. In total, 1175 images were acquired in both SWIR and VNIR spectral ranges, proving data for 3326 leaves in each case (Table 1).

**Table 1:** Number of images and leaves per treatment in SWIR and VNIR datasets.

Spectral range	Treatment	Number of hyperspectral images	Number of wheat leaves
SWIR	Infected	588	1652
	Non-infected	587	1674
	Total	1175	3326
VNIR	Infected	588	1652
	Non-infected	587	1674
	Total	1175	3326

### 4.3 Data analysis

**Fig. 6:**
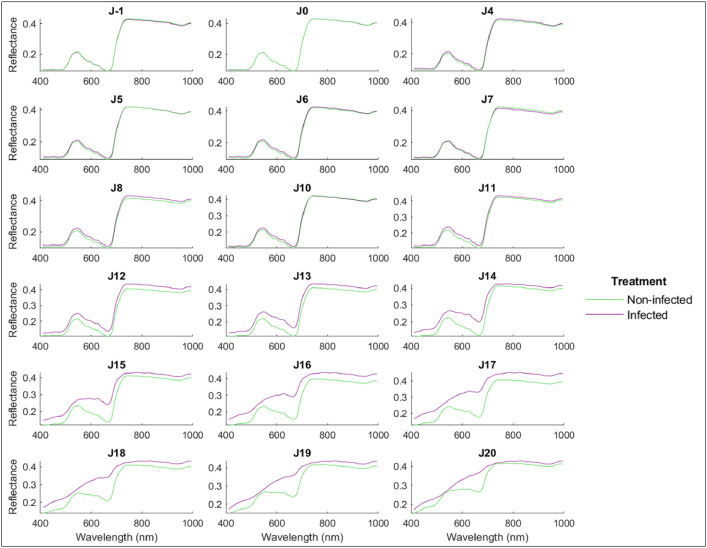
Average spectra of infected (purple) and non-infected (green) wheat leaves per day using VNIR spectral range.

**Fig. 7:**
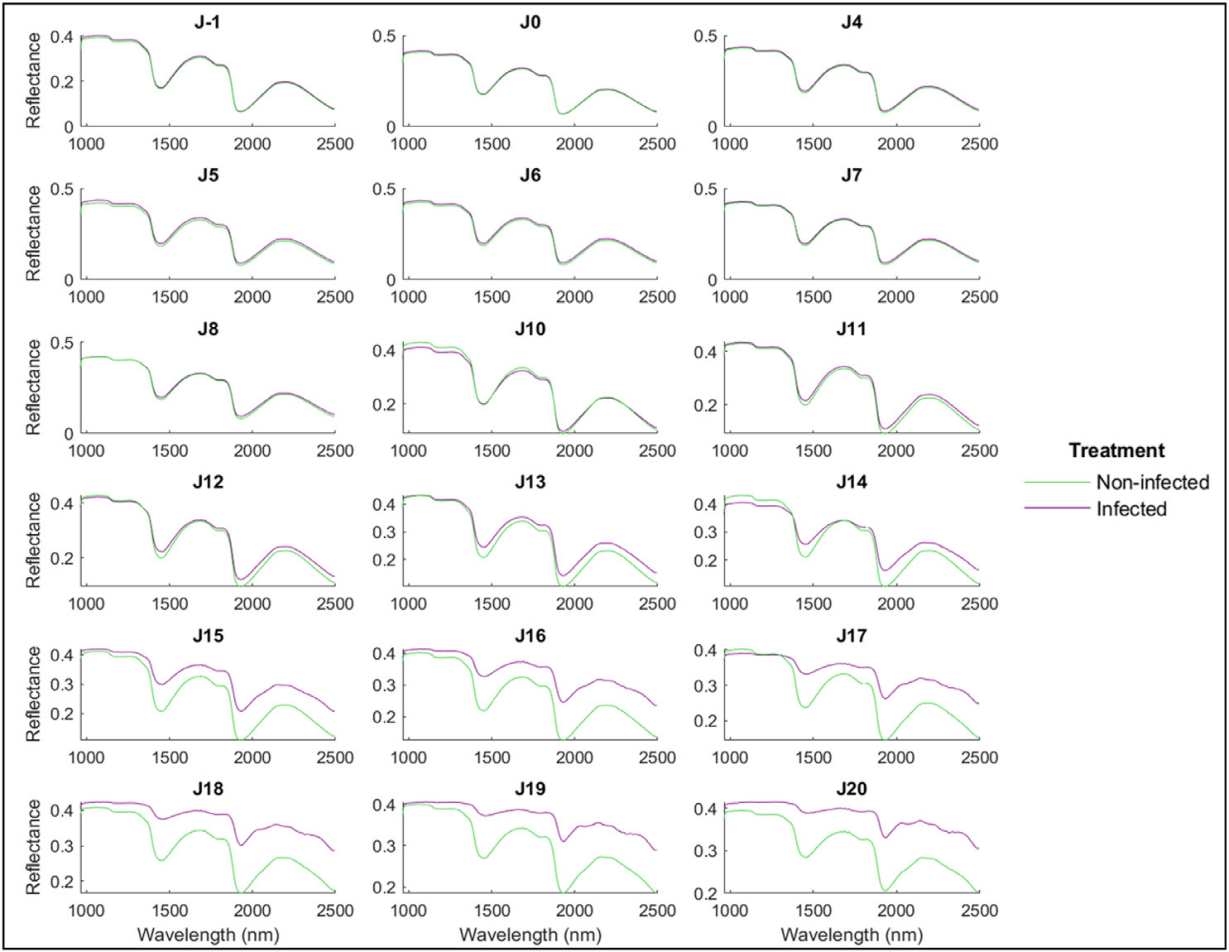
Average spectra of infected (purple) and non-infected (green) wheat leaves per day using SWIR spectral range.

HSI processing and multivariate analysis were performed under MATLAB® R2021a (The Mathworks, Natick, MA, USA) software. Images were reframed in order to focus on the inoculated area only, resulting in images of dimension 1370×781 pixels and 420×306 pixels for VNIR and SWIR images respectively. Pixels located on the inoculated leaf area were then extracted. Leaf pixel coordinates are summarized in (.csv) files and were used for spectral analysis. The average spectrum of each leaf was calculated, then the average spectra of infected and non-infected leaves per date were calculated and illustrated for VNIR (Fig. 6) and SWIR (Fig. 7) spectral ranges.

## Limitations

Plant senescence appeared earlier than expected, coinciding with the onset of symptoms. This might be explained by light conditions that accelerated the growth of wheat plants, thus reducing leaf life. It led to difficulties in assessing Lb disease, which can be confused with senescence. A confidence level of the disease was then set in place.

## Ethics Statement

The authors confirm that they have adhered to the ethical requirements for publication in Data in Brief. The present research study did not involve any human or animal subjects and did not use data collected from social media platforms.

## CRediT authorship contribution statement

**Lorraine Latchoumane:** Conceptualization, Formal analysis, Software, Visualization, Writing – original draft. **Martin Ecarnot:** Investigation, Methodology, Data curation, Writing – review & editing, Supervision. **Ryad Bendoula:** Investigation, Methodology, Data curation, Writing – review & editing, Supervision. **Jean-Michel Roger:** Writing – review & editing, Supervision. **Silvia Mas-Garcia:** Writing – review & editing, Supervision. **Heloïse Villesseche:** Investigation, Methodology, Data curation. **Flora Tavernier:** Investigation, Data curation. **Maxime Ryckewaert:** Writing – review & editing. **Nathalie Gorretta:** Supervision. **Pierre Roumet:** Investigation, Methodology, Data curation, Supervision. **Elsa Ballini:** Investigation, Methodology, Data curation, Writing – review & editing, Supervision.

## Data Availability

Data INRAEEarly detection of septoria infection on wheat leaves using hyperspectral imaging data (Original data)
